# Supplementary material for: Identification, Characteristics and Function of Phosphoglucomutase (PGM) in the Agar Biosynthesis and Carbon Flux in the Agarophyte Gracilariopsis lemaneiformis (Rhodophyta)
Source: Mar Drugs. 2022 Jul 2;20(7):442. doi: 10.3390/md20070442 (PMC9319447; doi:10.3390/md20070442)
Supplement: Supplementary file 1 [file marinedrugs-20-00442-s001.zip › marinedrugs-1777264-supplementary/supplementary materials/Fig S1.pdf]

A

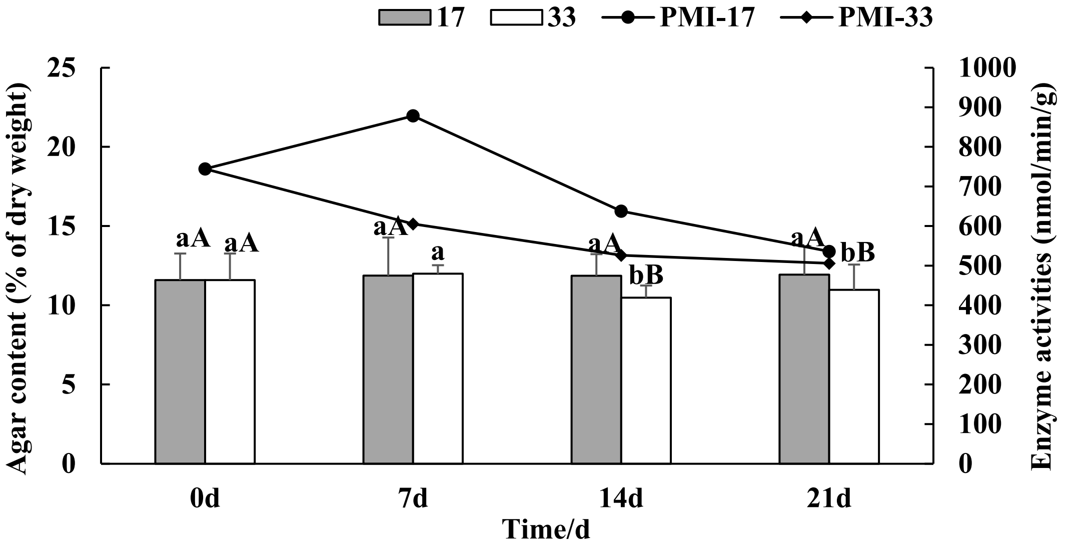

17
33
PMI-17
PMI-33
Agar content (% of dry weight)
Enzyme activities (nmol/min/g)
Time/d
0d
7d
14d
21d
aA
a
bB

B

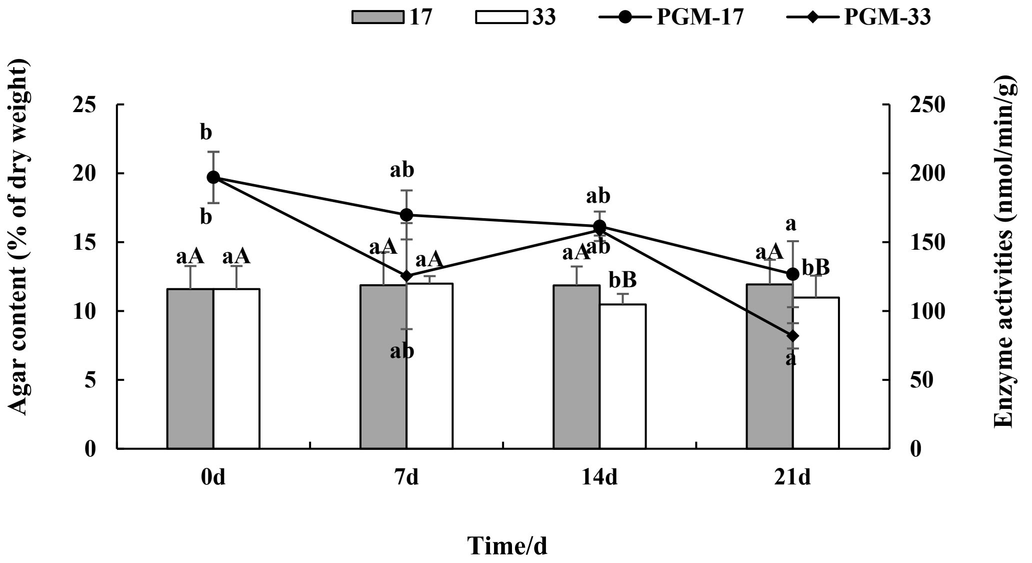

17
33
PGM-17
PGM-33
Agar content (% of dry weight)
Enzyme activities (nmol/min/g)
Time/d
0d
7d
14d
21d
aA
bB
ab
b
a

C

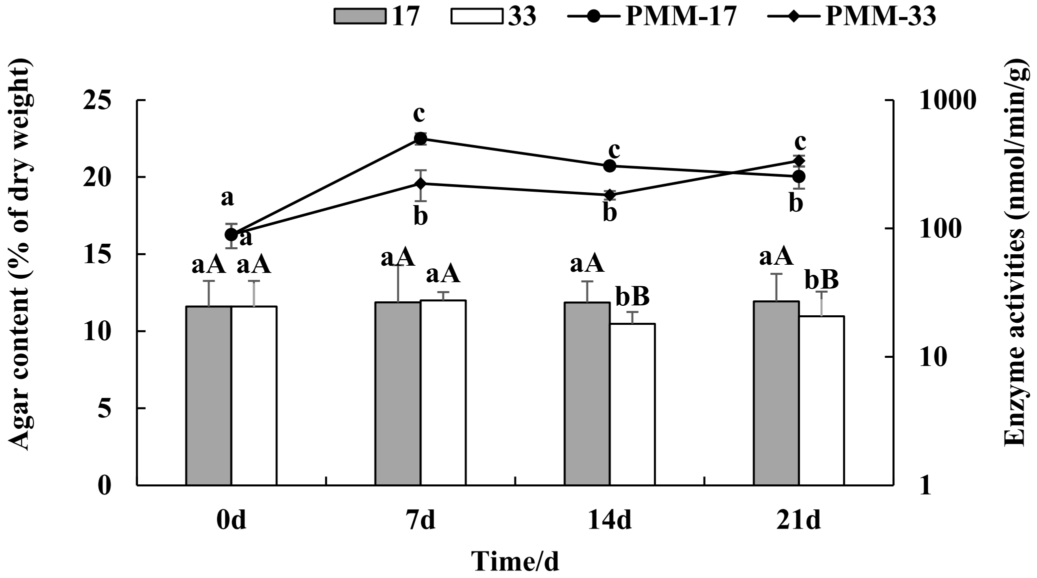

D

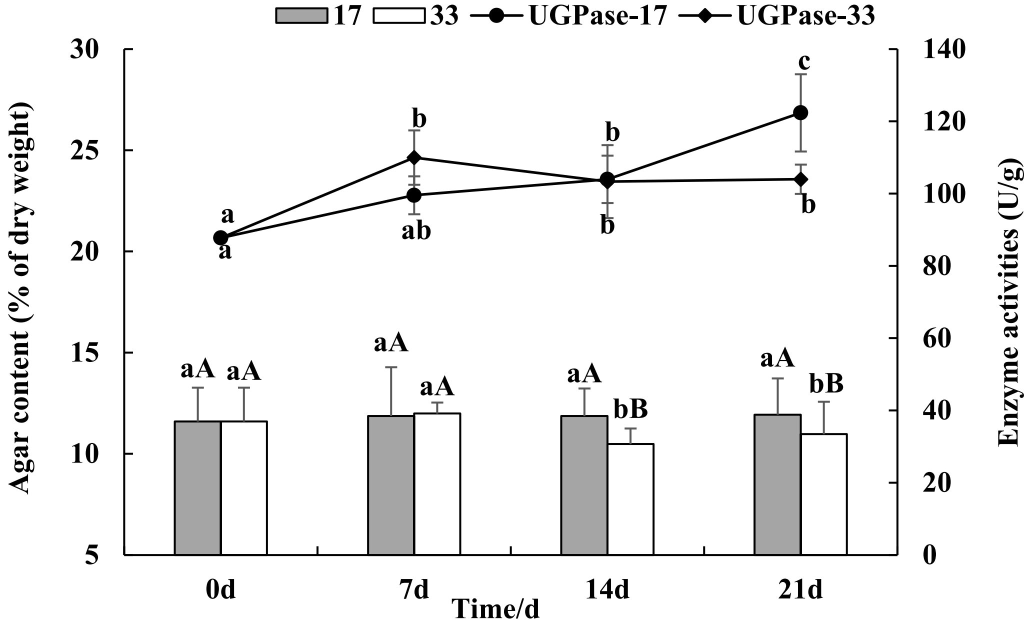

Figure S1 The changes of enzymic activities and agar contents in agar biosynthesis pathway in *Gracilariopsis lemaneiformis* in low (17) and normal (33) salinity treatments. A: PMI, mannose-6-phosphate isomerase; B: PGM, phosphoglucomutase; C: PMM, phosphomannomutase; D: UGPase,

UDP-glucose pyrophosphorylase. The lowercase and capital letters represent significant difference of agar contents and enzymic activities in different treatments, respectively ($p < 0.05$).
