# Supplementary material for: Identification, Characteristics and Function of Phosphoglucomutase (PGM) in the Agar Biosynthesis and Carbon Flux in the Agarophyte Gracilariopsis lemaneiformis (Rhodophyta)
Source: Mar Drugs. 2022 Jul 2;20(7):442. doi: 10.3390/md20070442 (PMC9319447; doi:10.3390/md20070442)
Supplement: Supplementary file 1 [file marinedrugs-20-00442-s001.zip › marinedrugs-1777264-supplementary/supplementary materials/Fig S2.pdf]

A

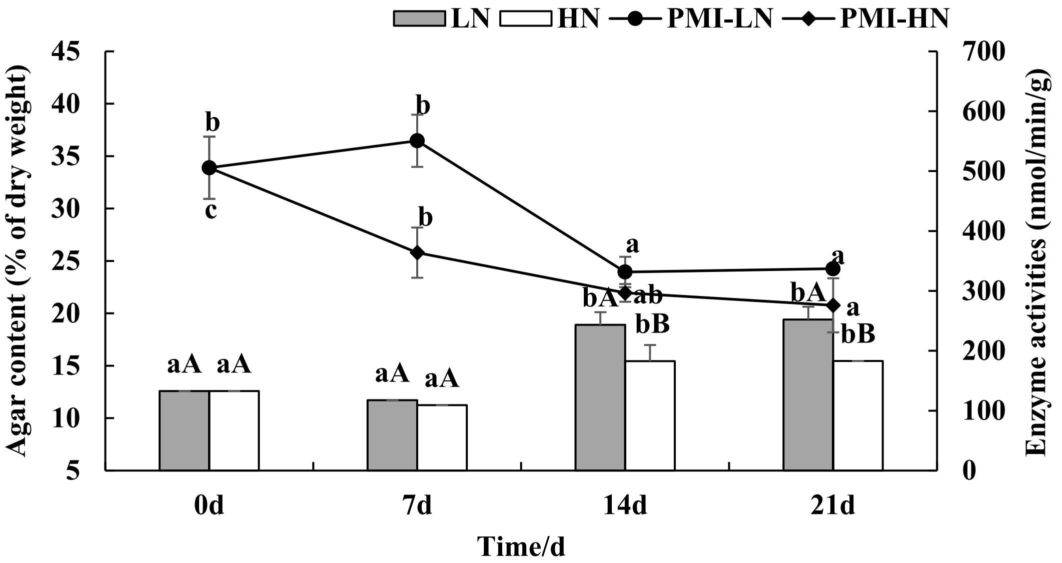
LN
HN
PMI-LN
PMI-HN
Agar content (% of dry weight)
Enzyme activities (nmol/min/g)
Time/d
0d
7d
14d
21d
aA
bA
bB
ab
a
b
c

B

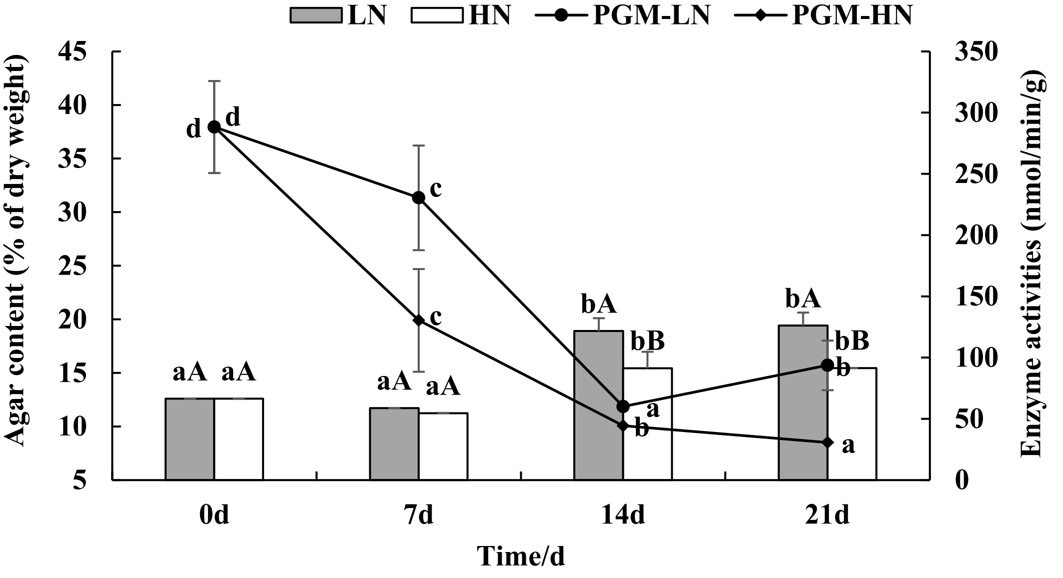
LN
HN
PGM-LN
PGM-HN
Agar content (% of dry weight)
Enzyme activities (nmol/min/g)
Time/d
0d
7d
14d
21d
aA
bA
bB
a
b
c
d

C

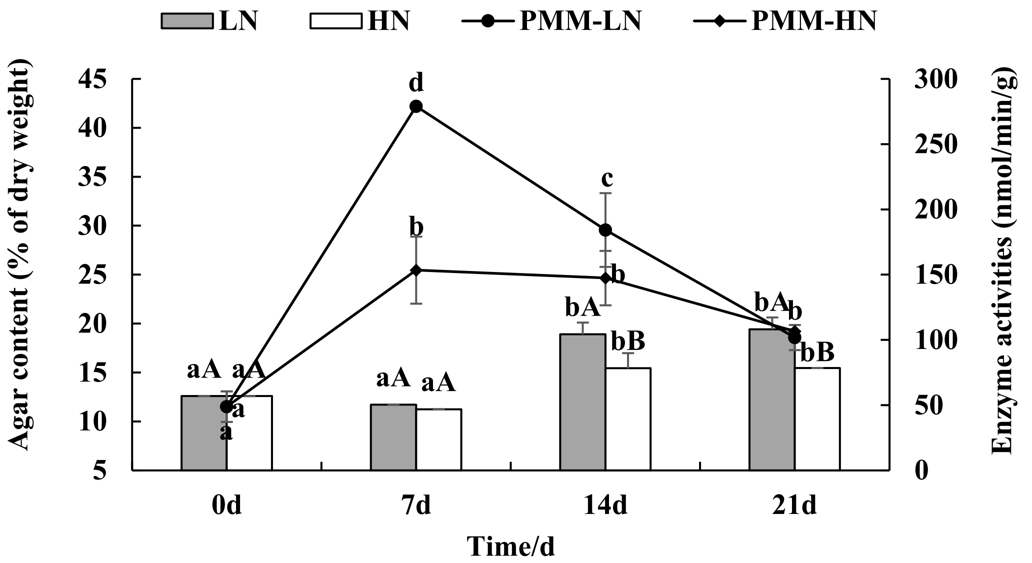

D

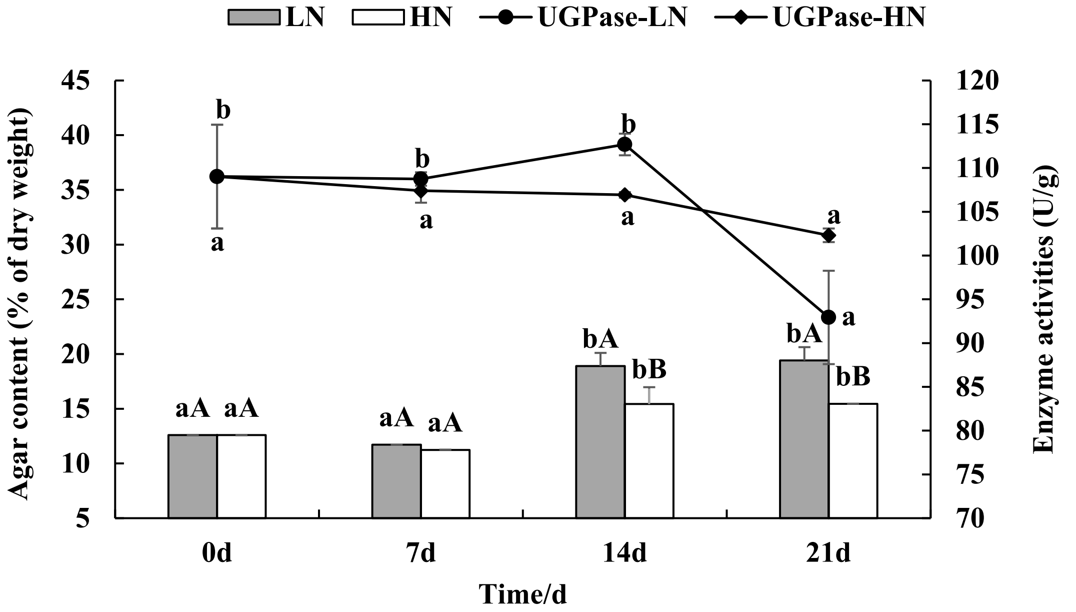

Figure S2 The changes of enzymic activities and agar contents in agar biosynthesis pathway in *Gracilariopsis lemaneiformis* in limited nitrogen (LN) and normal nitrogen (HN) treatments. A: PMI, mannose-6-phosphate isomerase; B: PGM, phosphoglucomutase; C: PMM, phosphomannomutase; D: UGPase, UDP-glucose pyrophosphorylase. The lowercase and capital letters represent significant difference of agar contents and enzymic activities in different treatments,

respectively ($p < 0.05$).
