# Supplementary material for: Identification, Characteristics and Function of Phosphoglucomutase (PGM) in the Agar Biosynthesis and Carbon Flux in the Agarophyte Gracilariopsis lemaneiformis (Rhodophyta)
Source: Mar Drugs. 2022 Jul 2;20(7):442. doi: 10.3390/md20070442 (PMC9319447; doi:10.3390/md20070442)
Supplement: Supplementary file 1 [file marinedrugs-20-00442-s001.zip › marinedrugs-1777264-supplementary/supplementary materials/Fig S3.pdf]

A

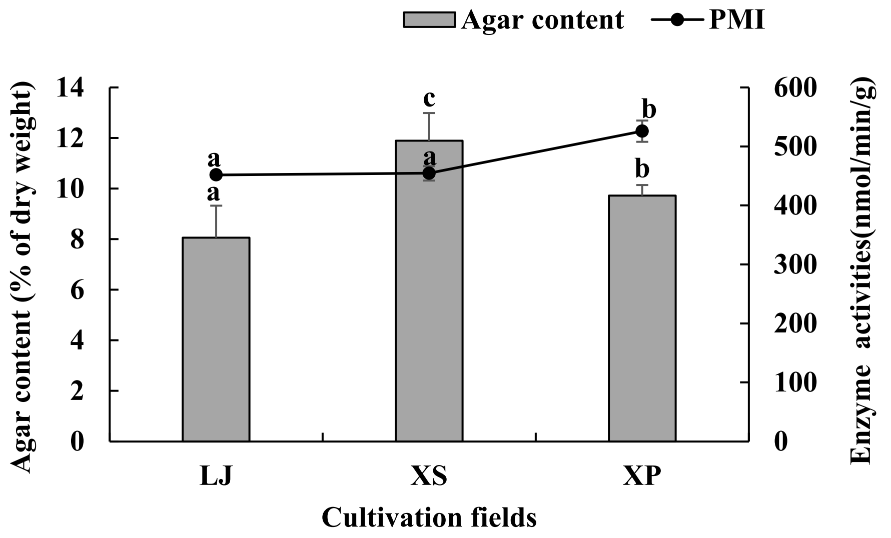
Agar content
PMI
Agar content (% of dry weight)
Enzyme activities(nmol/min/g)
14
12
10
8
6
4
2
0
600
500
400
300
200
100
0
a
a
c
a
b
b
LJ
XS
XP
Cultivation fields

B

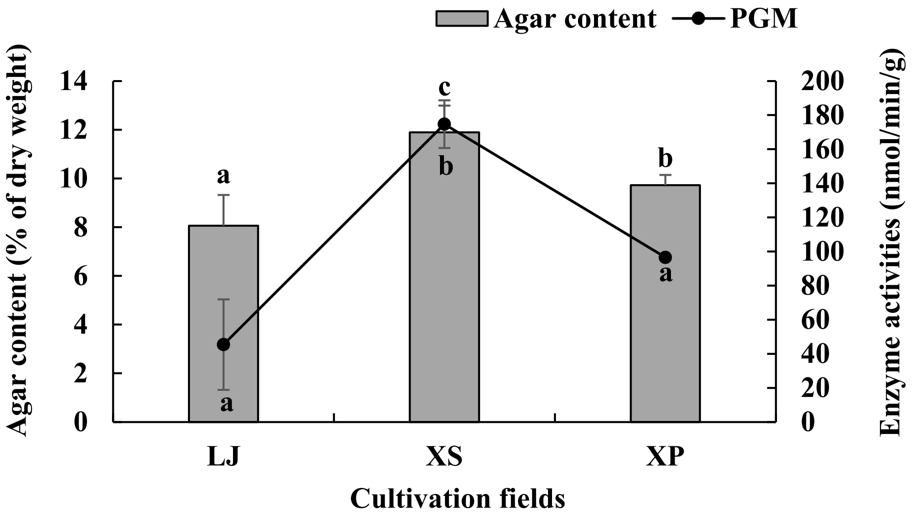
Agar content
PGM
Agar content (% of dry weight)
Enzyme activities (nmol/min/g)
14
12
10
8
6
4
2
0
200
180
160
140
120
100
80
60
40
20
0
a
a
c
b
b
a
LJ
XS
XP
Cultivation fields

C

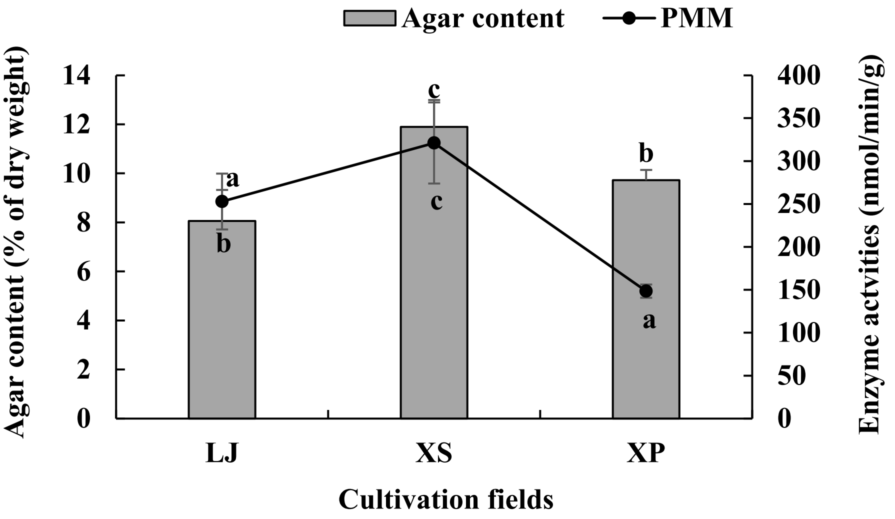

D

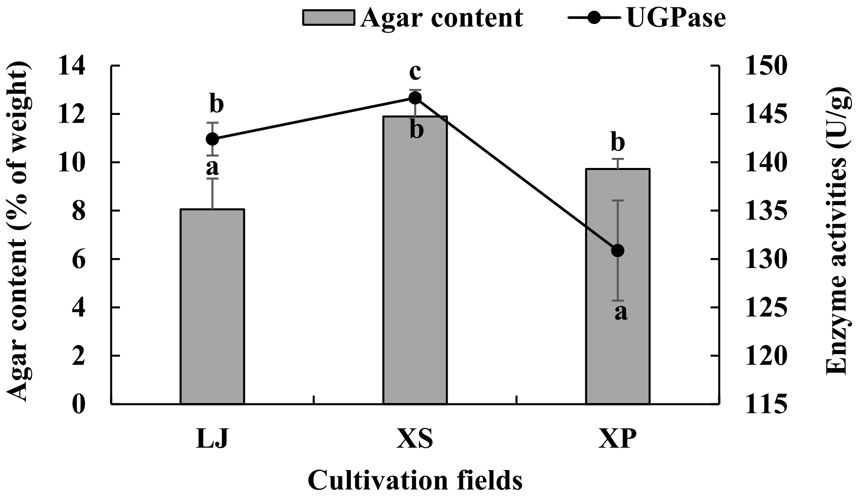

Figure S3 The changes of enzymic activities and agar contents in agar biosynthesis pathway in *Gracilariopsis lemaneiformis* in different cultivation fields. LJ, Lianjiang; XS, Xiangshan; XP, Xiapu. A: PMI, mannose-6-phosphate isomerase; B: PGM, phosphoglucomutase; C: PMM, phosphomannomutase; D: UGPase, UDP-glucose pyrophosphorylase. The lowercase and capital letters represent significant difference of agar contents and enzymic activities in different cultivation fields, respectively ($p < 0.05$).
