# Supplementary material for: Identification, Characteristics and Function of Phosphoglucomutase (PGM) in the Agar Biosynthesis and Carbon Flux in the Agarophyte Gracilariopsis lemaneiformis (Rhodophyta)
Source: Mar Drugs. 2022 Jul 2;20(7):442. doi: 10.3390/md20070442 (PMC9319447; doi:10.3390/md20070442)
Supplement: Supplementary file 1 [file marinedrugs-20-00442-s001.zip › marinedrugs-1777264-supplementary/supplementary materials/Fig S4.pdf]

A

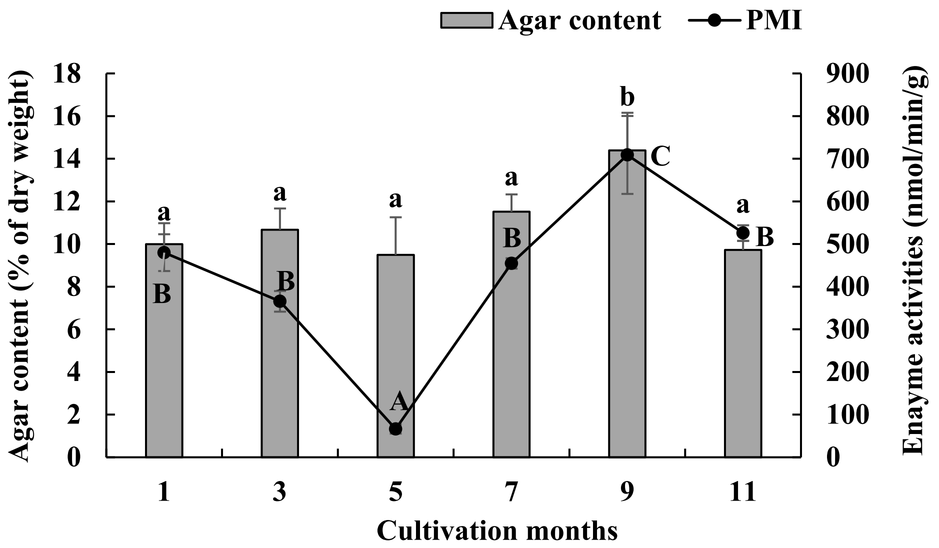
Agar content
PMI
Agar content (% of dry weight)
Enayme activities (nmol/min/g)
Cultivation months
18
16
14
12
10
8
6
4
2
0
900
800
700
600
500
400
300
200
100
0
1
3
5
7
9
11
a
a
a
a
b
a
B
B
A
B
C
B

B

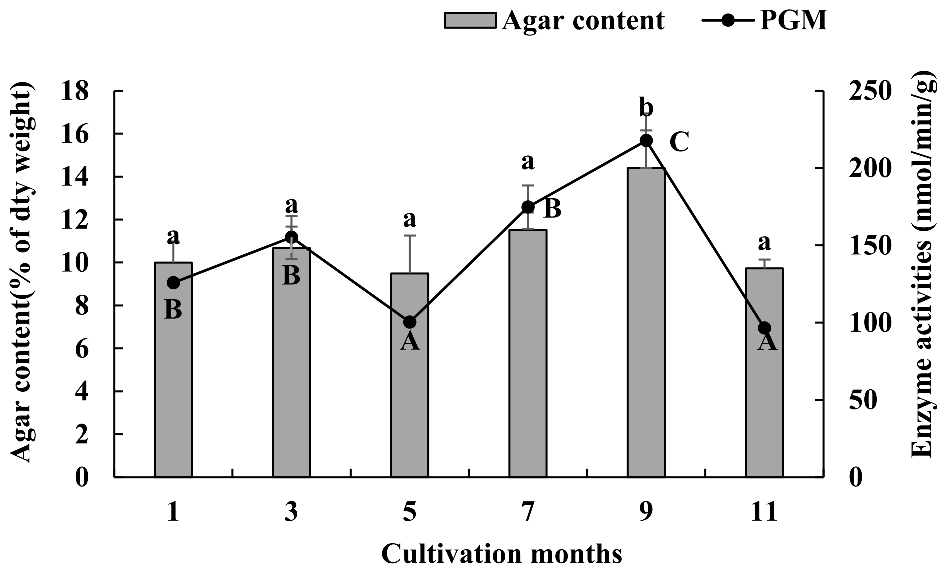
Agar content
PGM
Agar content(% of dty weight)
Enzyme activities (nmol/min/g)
Cultivation months
18
16
14
12
10
8
6
4
2
0
250
200
150
100
50
0
1
3
5
7
9
11
a
a
a
a
b
a
B
B
A
B
C
A

C

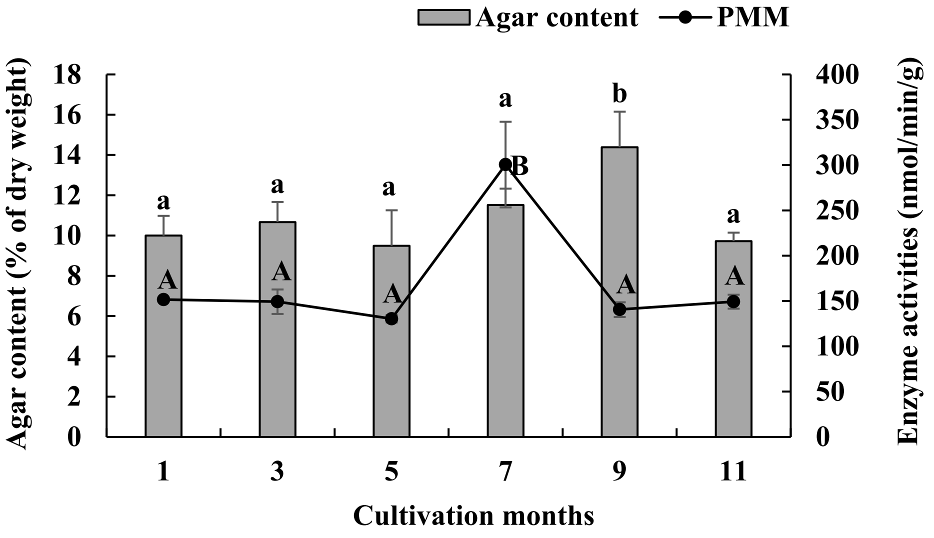

D

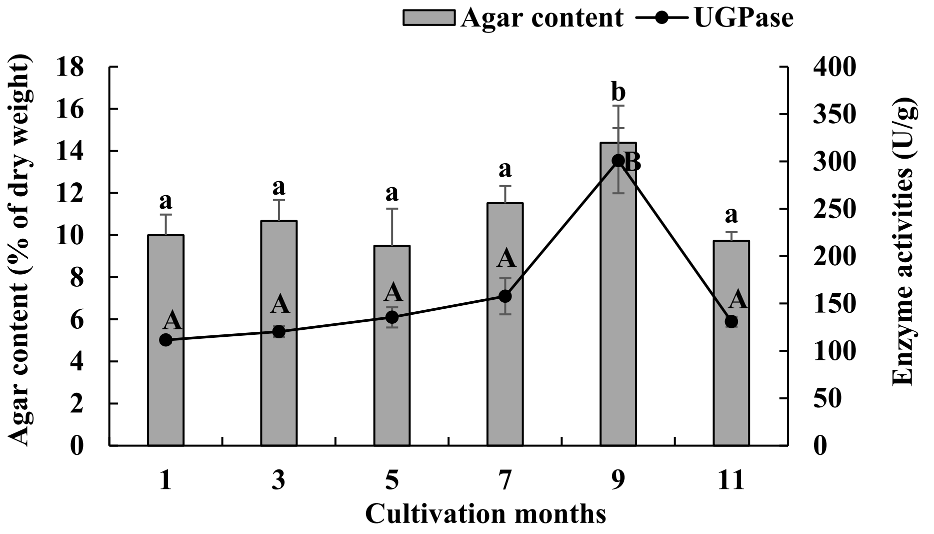

Figure S4 The changes of enzymic activities and agar contents in agar biosynthesis pathway in *Gracilariopsis lemaneiformis* in different cultivation months. A: PMI, mannose-6-phosphate isomerase; B: PGM, phosphoglucomutase; C: PMM, phosphomannomutase; D: UGPase, UDP-glucose pyrophosphorylase. The lowercase and capital letters represent significant difference of agar contents and enzymic activities in different cultivation months, respectively ($p < 0.05$).
