# Supplementary material for: Identification, Characteristics and Function of Phosphoglucomutase (PGM) in the Agar Biosynthesis and Carbon Flux in the Agarophyte Gracilariopsis lemaneiformis (Rhodophyta)
Source: Mar Drugs. 2022 Jul 2;20(7):442. doi: 10.3390/md20070442 (PMC9319447; doi:10.3390/md20070442)
Supplement: Supplementary file 1 [file marinedrugs-20-00442-s001.zip › marinedrugs-1777264-supplementary/supplementary materials/Fig S5.pdf]

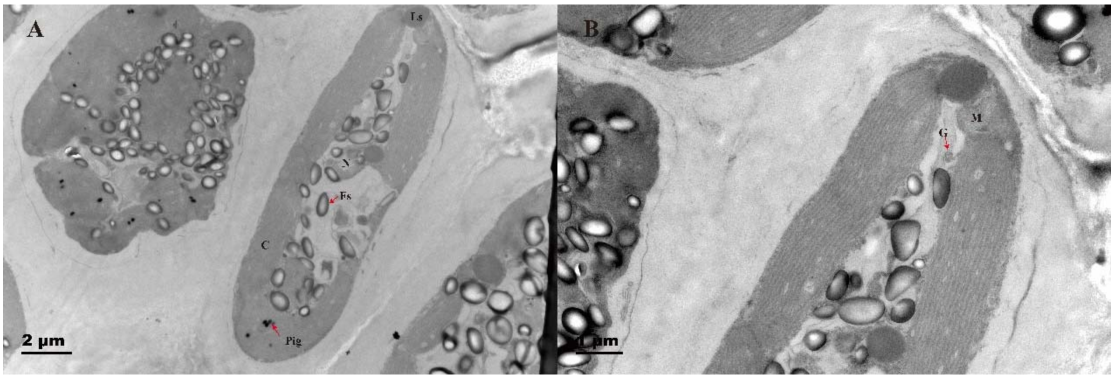

Figure S5 The ultrastructure of *Gracilariopsis lemaneiformis* under transmission electron microscope (TEM). A: ultrastructure of *G. lemaneiformis* at the scale of 2 μM; B: ultrastructure of *G. lemaneiformis* at the scale of 1 μM. C, chloroplast; N, nuclear; Fs, floridean starch; Pig, pigment; Ls, liposome; G, Glogi complex; M, mitochondria.
