# Supplementary material for: Identification, Characteristics and Function of Phosphoglucomutase (PGM) in the Agar Biosynthesis and Carbon Flux in the Agarophyte Gracilariopsis lemaneiformis (Rhodophyta)
Source: Mar Drugs. 2022 Jul 2;20(7):442. doi: 10.3390/md20070442 (PMC9319447; doi:10.3390/md20070442)
Supplement: Supplementary file 1 [file marinedrugs-20-00442-s001.zip › marinedrugs-1777264-supplementary/supplementary materials/Fig S6.pdf]

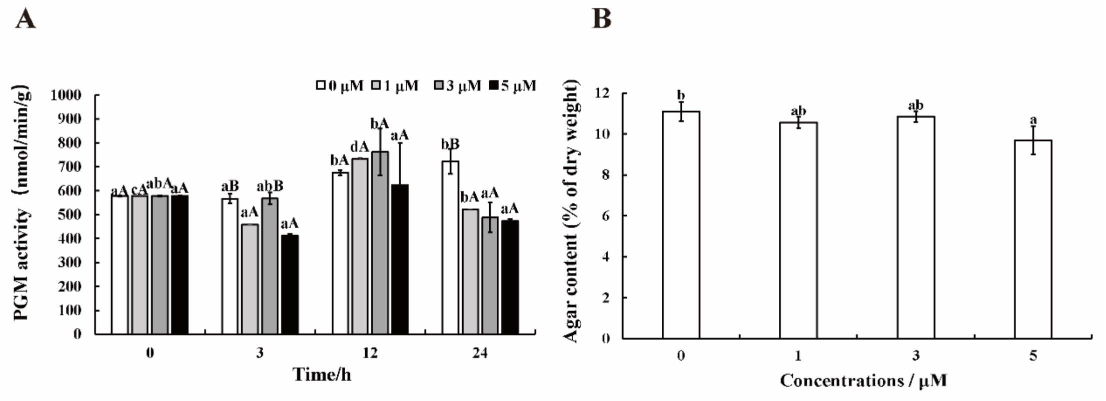

Figure S6 Phosphoglucomutase (PGM) activities and agar contents of *Gracilariopsis lemaneiformis* under PGM inhibited condition. A: PGM activities under different PGM inhibitor concentrations within 24 h. B: agar contents under different PGM inhibitor concentrations at 24 h. The lowercase letters represent significant difference of the treatment with time, and capital letters represent significant differences between different treatments ($p < 0.05$).
