# Supplementary material for: Identification, Characteristics and Function of Phosphoglucomutase (PGM) in the Agar Biosynthesis and Carbon Flux in the Agarophyte Gracilariopsis lemaneiformis (Rhodophyta)
Source: Mar Drugs. 2022 Jul 2;20(7):442. doi: 10.3390/md20070442 (PMC9319447; doi:10.3390/md20070442)
Supplement: Supplementary file 1 [file marinedrugs-20-00442-s001.zip › marinedrugs-1777264-supplementary/supplementary materials/Fig S7.pdf]

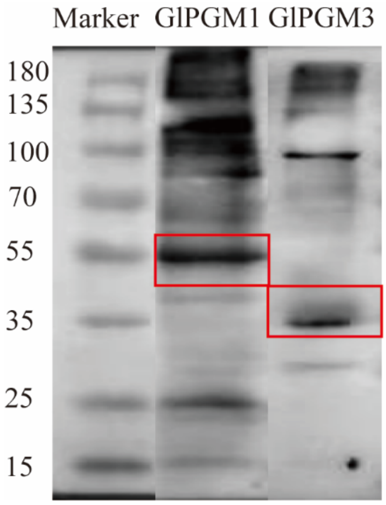

Figure S7 Western blot analysis of GlPGM1 and GlPGM3 in *Gracilariopsis lemaneiformis*
Red boxes indicate the GlPGM1 and GlPGM3 proteins.
