# Supplementary material for: Identification, Characteristics and Function of Phosphoglucomutase (PGM) in the Agar Biosynthesis and Carbon Flux in the Agarophyte Gracilariopsis lemaneiformis (Rhodophyta)
Source: Mar Drugs. 2022 Jul 2;20(7):442. doi: 10.3390/md20070442 (PMC9319447; doi:10.3390/md20070442)
Supplement: Supplementary file 1 [file marinedrugs-20-00442-s001.zip › marinedrugs-1777264-supplementary/supplementary materials/Table S1.pdf]

Table S1 PCR information for the amplification and cloning of *GlPGM1*, *GlPGM2* and *GlPGM3*

Primers	Sequence (5'-3')	restriction endonuclease	Tm (℃)	PCR products
Glpgm1-ORF5'	CGCGAATTCATGGCTTCGACTTTCAAGGTC	*EcoR* I	62	1758bp
Glpgm1-ORF3'	CGCAAGCTTTTAAGTGATAACTGTGGGCTTC	*Hind* III		
Glpgm2-ORF5'	CGCGAGCTCATGAGCCAGATCCCCCAATC	*Sac*I	58	1743bp
Glpgm2-ORF3'	CGCCTCGAGTTAAGTAATCACTGTGGGAGC	*Xho* I		
Glpgm3-ORF5'	CGCGAATTCATGTACTCCTTCATCACCTCC	*EcoR* I	65	1719bp
Glpgm3-ORF3'	CGCAAGCTTTTAATTCTTCTGAATGTATTGCTGC	*Hind* III		

The underline represents restriction endonuclease sequence.
