# Supplementary material for: Identification, Characteristics and Function of Phosphoglucomutase (PGM) in the Agar Biosynthesis and Carbon Flux in the Agarophyte Gracilariopsis lemaneiformis (Rhodophyta)
Source: Mar Drugs. 2022 Jul 2;20(7):442. doi: 10.3390/md20070442 (PMC9319447; doi:10.3390/md20070442)
Supplement: Supplementary file 1 [file marinedrugs-20-00442-s001.zip › marinedrugs-1777264-supplementary/supplementary materials/Table S2.pdf]

Table S2 The information of phosphoglucomutase (PGM) protein sequences from genome of different species

Taxonomy	Species	Gene ID
Arabidopsis thaliana	Spermatophyta	NP_199995.1<br>NP_173732.1<br>NP_177230.1<br>NP_001154464.1<br>NP_001154465.1<br>NP_001320476.1<br>NP_177239.1<br>NP_001332305.1<br>NP_001318587.1<br>NP_001190326.1<br>NP_001332304.1<br>NP_568359.2
Gossypium raimondii	Spermatophyta	XP_012467251.1<br>XP_012467267.1<br>XP_012454357.1<br>XP_012454356.1<br>XP_012454355.1<br>XP_012454353.1<br>XP_012467252.1<br>XP_012440230.1<br>XP_012459882.1
Oryza sativa	Spermatophyta	XP_015614961.1<br>XP_015632142.1<br>XP_015643632.1<br>XP_015647517.1<br>XP_015647516.1<br>XP_015643633.1
Zea mays	Spermatophyta	XP_008675355.1<br>NP_001355137.1<br>NP_001105405.1<br>NP_001105703.1<br>XP_008652327.1<br>XP_008652325.1<br>XP_008673528.1<br>XP_035816183.1<br>XP_020395615.1<br>XP_020395614.2
Chlamydomonas reinhardtii	Chlorophyta	XP_001689884.1<br>XP_001697992.1

Micromonas pusilla	Chlorophyta	XP_001697991.1
		XP_001695635.1
		XP_003056074.1
		XP_003056559.1
Ostreococcus lucimarinus CCE9901	Chlorophyta	XP_001421345.1
		XP_001415757.1
Aureococcus anophagefferens	Ochrophyta	XP_009036707.1
		XP_009035725.1
Ectocarpus siliculosus	Ochrophyta	CBJ31711.1
		CBN79734.1
		CBJ48481.1
		CBJ29496.1
Phaeodactylum tricornutum	Ochrophyta	Phatr3_EG02613.p1
		Phatr3_J32708.p1
		Phatr3_J16571.p1
		Phatr3_EG02240.p1
Gracilariopsis chorda	Rhodophyta	PXF45253.1
		PXF46312.1
		PXF42557.1
		PXF44087.1
Chondrus crispus	Rhodophyta	XP_005717610.1
		XP_005713583.1
		XP_005717288.1
Cyanidioschyzon merolae strain 10D	Rhodophyta	XP_005538026.1
		XP_005539294.1
		XP_005534981.1
		XP_005535333.1
Porphyra umbilicalis	Rhodophyta	OSX75697.1
		OSX73648.1
		OSX71554.1
		OSX69066.1
Porphyridium purpureum	Rhodophyta	evm.model.contig_2285.4
		evm.model.contig_452.2
		evm.model.contig_3397.5
		evm.model.contig_4446.7
Phytophthora sojae	Stramenopiles	XP_009532079.1
		XP_009514793.1
Talaromyces islandicus	Ascomycota	CRG86074.1
		CRG91341.1
		CRG92507.1
Microsporum canis	Ascomycota	XP_002850691.1
		XP_002843246.1
Trichormus variabilis	Cyanobacteria	WP_011320468.1
		WP_011318541.1

Caldivirga maquilingensis	Archaea	WP_011319151.1<br>WP_011317071.1<br>WP_156769897.1<br>WP_048063059.1
